# Brave (in a) new world: an ethical perspective on chatbots for medical advice

**DOI:** 10.3389/fpubh.2023.1254334

**Published:** 2023-08-17

**Authors:** Thomas C. Erren, Philip Lewis, David M. Shaw

**Affiliations:** ^1^University of Cologne, University Hospital of Cologne, Cologne, North Rhine-Westphalia, Germany; ^2^Care and Public Health Research Institute, Maastricht University, Maastricht, Netherlands; ^3^Institute for Biomedical Ethics, University of Basel, Basel, Switzerland

**Keywords:** chatbot, ChatGPT, medical advice, ethics, confidentiality and privacy, risks, hallucination

“*Words can be like X-rays if you use them properly—they'll go through anything. You read and you're pierced.”*
-Aldous Huxley, Brave New World (1).

## 1. Introduction

Huxley's dystopian novel *Brave New World* (1) describes a futuristic World State with immense scientific advances, but also psychological manipulation and classical conditioning. Today's “free-to-use” ChatGPT, which is taking the world by storm, should lead to discussions about the disruptive impact that artificial intelligence (AI) could have on our future, how to shape it, and how to avoid dystopian developments. Indeed, AI is an important frontier in public health that requires ethical discussion (2).

ChatGPT is a large language model (LLM) trained with very large amounts of textual data to generate new texts in response to text prompts from humans. Its responses resemble human answers to human questions. The progress and success of this deep learning model has also puzzled its developers at OpenAI (San Francisco, CA) (3). Both concerns and possibilities (4–9) have been raised about the impact and developments of ChatGPT or similar AI.

Like a tsunami, the emergence of chatbots sweeps us into *terra incognita*. More waves of AI will follow. In such a climate, our opinion targets chatbots to contribute to medical advice from ethical points of view. By medical advice [MA] we mean integrated, private, confidential, dependable, and trustworthy health and medical information for citizens. Numerous articles from 2023 deal with ChatGPT and medicine and MA, but few from the perspectives of ethics (2, 5–7, 10–14).

ChatGPT's ability to provide on-demand and specific answers to questions could surpass the use of “Dr Google” when searching for medical and health-related information (15–17). While “Dr Google” returns torrents of information that citizens must wade through, “Dr ChatGPT” offers users more focused distillations, although the results may still not be accurate.

Considering that chatbots mimic conversational interaction, we ask: What could come next? Where can AI take us, possibly faster than most expect? What can we do? And what should we do? In the following sections, we outline current warnings of chatbots like ChatGPT from developers and calls for ethical discourse. In regards to MA, we sketch potential developments of chatbots and associated risks, hallucinations, and “bullshit.” From an ethics perspective, we address the critical confidentiality of information and data, which serve as key drivers of advancing AI, and close with imperative questions and guardrails to benefit from chatbots and avoid dystopian developments.

## 2. Current warnings and calls for ethical discourse

A powerful call for ethical discourse came on May 30, 2023, in a one-sentence statement signed by more than 350 AI executives, researchers, and engineers: “Mitigating the risk of extinction from AI should be a global priority alongside other societal-scale risks such as pandemics and nuclear war” (18). On May 16, 2023 (19), the OpenAI CEO Altman urged for regulation of AI in a US Senate panel hearing: “We think that regulatory intervention by governments will be critical to mitigate the risks of increasingly powerful models.” Moreover, “‘The Godfather of AI' leaves Google and warns of danger ahead” wrote the NYT on May 1, 2023 (20): “For half a century, Geoffrey Hinton nurtured the technology at the heart of chatbots like ChatGPT. Now he worries it will cause serious harm.”

Clearly, even developers foresee massive potential for disruption by AI technology. Thus, the question arises as to how we can “prioritise responsible and beneficial applications that serve the best interests of society” (13), including utmost reliability, privacy, confidentiality, data protection, and disclosure of AI interests? We think that we should have ethical debate and develop safe-guards and red lines to allow the good and disallow the bad—and given the stakes, to initially err on the side of caution.

## 3. Chatbot developments

One evolutionary step for chatbots like ChatGPT is that “chatting,” which today consists of typing and reading, will become talking and listening. That the voice serves as the AI control and response device, would be in line with the HAL 9000 computer in the science fiction classic “2001: A Space Odyssey.” In that film, HAL (Heuristically programmed ALgorithmic computer) is an advanced AI, programmed to obey and not harm its creators, and should respond to voice instructions of the human crew when controlling their spaceship Discovery One.

Such technically advanced advice service seems neither far-fetched nor distant. *Alexa, Siri*, and *Cortana*, produced by Amazon, Apple, and Microsoft, respectively, are already voice-activated and voice-responding internet-connected devices as part of the “Internet of Things (IoTs).” Combined with spoken language systems, citizens will talk and listen to chatbots that combine extensive (personal and general) information with massive computing power. Unlike ChatGPT, which has been closed off from new information since September 2021 (7), advanced AI for MA would have access to real-time information in order to provide up-to-date MA.

## 4. Risks, hallucinations, and “bullshit”

What about potential risks to citizens—both patients and doctors—who become “information providers and consumers”? What about potential mind-manipulation—be that intentional or unintentional—of citizens through convincingly worded and reasoned individualized advice? With Huxley's *Brave New World* in mind, is it possible that the boundaries between human and machine will become so blurred that citizens will no longer be able to distinguish MA provided by chatbots from that given by humans? Or might they not recognize who they give their personal information to? Could weakened encryption of human-machine exchanges reduce individuals' control over data (21) and open doors to ethically unethical surveillance?

While made-up “facts” or hallucinations in AI (7) limit ChatGPT's results in relation to science, representatives of medicine are beginning to weigh its potential utility and benefits for numerous areas and applications. But: In view of the broad interest in ChatGPT, please bear a key point in mind: despite extensive media coverage stating the contrary, ChatGPT is not capable of human levels of thought. It is a sophisticated chatbot that is trained on vast quantities of data to offer persuasively sounding responses. Sometimes these responses are accurate; sometimes they are not. Sometimes its rhetoric is so persuasive that gaps in logic and facts are obscured. In effect, ChatGPT includes the generation of “bullshit” (22, 23) i.e., speech intended to persuade but without regard for truth, and such “bullshit” can be right some of the time. The question is whether citizens should seek MA from such a fallible information source.

## 5. Critical confidentiality: information and data = key drivers of AI advances

The current lack of information about how personal data is “used” makes AI boxes opaque: Are citizens aware of this non-transparent use and what control is in place so that personal data is not shared and disseminated for uses beyond MA? A key driver is that the more information citizens provide to AI, the more personalized (and potentially better) MA can become. However, this could lead patients and doctors to provide ever more information at the expense of privacy and confidentiality, making citizens and their data unduly transparent; thereby, potentially opening the door to other uses of their data.

In a nutshell, the *modus operandi* of current chatbot success is that “Artificial intelligence could never have been so successful in recent years … if these corporations had not collected masses of data. This information enabled them to train their AI models in the first place. This—in addition to an increase in computing power—is the driver of the current AI boom” (24).

## 6. Imperative questions from an ethics perspective

That society will not abandon the potential of LLMs is a realistic prospect. Under this assumption, the following questions (Box) should be urgently discussed in order to consider the mindful use of AI for MA. The answers to these questions seem open, but they must be found, and quickly.

Box 1 **Imperative questions for the mindful use of AI for medical advice [MA].**

**Regulation of AI**

• Who “programs and controls” AI and “how,” i.e., with what interests: what biases result for MA?

**Control of personal information**

• Who protects the information that AI collects from individual citizens and through doctors, and how?
• How will potential use (“sharing”) of information for purposes other than the requested MA be regulated or ruled out? For instance: How can we safeguard patients' information from commercial exploitation (e.g., the generation of MA were to be misused as a Trojan horse for commercial advantages)?

**MA & the role of doctors**

• How can we deal with MA for which AI cannot provide explanations as to how it was arrived at? (25).
• Medical knowledge—which chatbots will have more of than a doctor at any given time—does not equate to quality of MA: What are doctors' roles in reviewing, monitoring, and controlling MA by AI?
• Can doctors become biased by AI-provided diagnoses and AI-suggested treatments such that they miss true causes and more appropriate therapies of ill-health? In other words, could they become over-reliant on AI?
• Could it be that doctors who do not use AI such as ChatGPT may give less than adequate information and advice and could such doctors be accused of providing substandard care? (25, 26).
• What knowledge and how much time do doctors need to invest to understand MA via AI and when can they use or endorse AI recommendations and with how much confidence?
• Human decisions may be badly influenced by information provided by chatbots: What are doctors' roles in regards to scrutinize and maintain control over MA via AI?

**Liability**

• Who is liable for MA via AI as an available resource (26, 28)?
• Who is liable when doctors use (or ignore) MA via AI? (25)

**Regarding all of the above**

• Who should set which boundaries and how and when?

To exemplify the complexities above, let us briefly look at liability. When and how doctors who use medical AI could be held liable under current law is explored step-by-step elsewhere (25). Because AI is new to medical practice in general and to medical advice in particular, and with the lack of case law on liability when using AI, physicians would be entering *terra incognita*. To offer orientation, Price et al. (25) took the following approach: with a view to more general principles of tort law, examples of likely or potential legal consequences of the use of AI in clinical practice were developed. Importantly, the current legal basis for liability for medical AI, in which MA can play a central role, is unlikely to remain unchanged. As a rule of thumb, whenever AI is used to replace human (clinician) judgment, this may pose safety risks to patients and may render clinicians legally liable (26).

## 7. Ethical guardrails to benefit from chatbots and avoid dystopian developments

The ever-evolving chatbots have the potential to benefit us in personalized ways, but they also have the potential to manipulate and condition us through effective words and language. As for the power of information and data, they are the fuel for the performance and ultimately the competence of chatbots. As one step toward remedying conceivable misuse of information, the publication practice that authors disclose all possible conflicts of interest should also apply to AI and the companies that develop such products. But: Shouldn't we collect, store, connect, and share information about citizens as little as possible—and if at all, then anonymized and encrypted?

Overall, we have outlined current warnings from AI developers, sketched potential developments and associated risks of using chatbots regarding MA, and provided imperative ethical questions. As humans are unlikely to forego the use of AI, significant ethical challenges need to be addressed. Echoing the cautionary tale in the introduction, we need to guard against bias, protect trust, equality and privacy, and establish a “Code of Conduct for AI in Health Care” (11) and guidelines for MA.

Of course, we should do all we ethically can to benefit from chatbot advice, provided it is medically sound. It is equally clear that we must avoid the danger of Orwellian transparency (27) and of conditioning (Huxley's “mind-manipulation”) to believe in non-sensical information about our bodies and health and in non-sensical MA. The latter would be a recipe for not having to be brave in the new AI world that lies ahead.

## Author contributions

TE: Conceptualization, Writing—original draft, Writing—review and editing. PL: Writing—review and editing. DS: Writing—review and editing.

## References

[1] Huxley A. Brave New World. London: Chatto and Windus (1932).

[2] De Angelis L Baglivo F Arzilli G Privitera GP Ferragina P Tozzi AE. ChatGPT and the rise of large language models: the new AI-driven infodemic threat in public health. Front Public Health. (2023) 11:1166120. 10.3389/fpubh.2023.1166120 37181697 PMC10166793

[3] Heaven WD. Artificial Intelligence—The Inside Story of How ChatGPT Was Built From the People Who Made it. (2023). Available online at: https://www.technologyreview.com/2023/03/03/1069311/inside-story-oral-history-how-chatgpt-built-openai/ (accessed 7.5.2023).

[4] Brainard J. Journals take up arms against AI-written text. Science. (2023) 379:740–1. 10.1126/science.adh2762 36821673

[5] Chow JCL Sanders L Li K. Impact of ChatGPT on medical chatbots as a disruptive technology. Front Artif Intell. (2023) 6:1166014. 10.3389/frai.2023.1166014 37091303 PMC10113434

[6] Dave T Athaluri SA Singh S. ChatGPT in medicine: an overview of its applications, advantages, limitations, future prospects, ethical considerations. Front Artif Intell. (2023) 6:1169595. 10.3389/frai.2023.1169595 37215063 PMC10192861

[7] Shaw D Morfeld P Erren T. The (mis)use of ChatGPT in science and education: turing, Djerassi, “athletics” and ethics. EMBO Rep. (2023) 23:e57501. 10.15252/embr.202357501 37259767 PMC10328063

[8] Stokel-Walker C Van Noorden R. What ChatGPT and generative AI mean for science. Nature. (2023) 614:214–6. 10.1038/d41586-023-00340-6 36747115

[9] Thorp HH. ChatGPT is fun, but not an author. Science. (2023) 379:313. 10.1126/science.adg7879 36701446

[10] Beltrami EJ Grant-Kels JM. Consulting ChatGPT: ethical dilemmas in language model artificial intelligence. J Am Acad Dermatol. (2023) 2:52. 10.1016/j.jaad.2023.02.052 36907556

[11] Dorr DA Adams L and Embi P. (2023). Harnessing the Promise of Artificial Intelligence Responsibly. JAMA. 329:1347–8. 10.1001/jama.2023.2771 36972068

[12] Kavian JA Wilkey HL Patel PA Boyd JC. Harvesting the power of artificial intelligence for surgery: uses, implications, ethical considerations. Am Surg. (2023) 23:31348231175454. 10.1177/00031348231175454 37148260

[13] Li H Moon JT Purkayastha S Celi LA Trivedi H Gichoya WJ. Ethics of large language models in medicine and medical research. Lancet Digit Health. (2023) 23:83. 10.1016/S2589-7500(23)00083-3 37120418

[14] Marchandot B Matsushita K Carmona A Trimaille A Morel O. ChatGPT: the next frontier in academic writing for cardiologists or a pandora's box of ethical dilemmas. Eur Heart J Open. (2023) 3:oead007. 10.1093/ehjopen/oead007 36915398 PMC10006694

[15] Lam-Po-Tang J McKay D. Dr Google, MD: a survey of mental health-related internet use in a private practice sample. Australas Psychiatr. (2010) 18:130–3. 10.3109/10398560903473645 20175669

[16] Hyman I,. The Risks of Consulting Dr. Google. Googling Information Can Cause Harm Anxiety, Especially in a Pandemic. (2020). Available online at: https://www.psychologytoday.com/us/blog/mental-mishaps/202004/the-risks-consulting-dr-google (accessed 23.5.2023).

[17] Van Bulck L Moons P. What if your patient switches from Dr. Google to Dr. ChatGPT? a vignette-based survey of the trustworthiness, value and danger of ChatGPT-generated responses to health questions. Eur J Cardiovasc Nurs. (2023) 23:zvad038. 10.1093/eurjcn/zvad038 37094282

[18] Center for AI safety. Statement on AI Risk. AI experts and public figures express their concern about AI risk (2023). Available online at: https://www.safe.ai/statement-on-ai-risk (accessed June 20, 2023).

[19] Fung B,. (2023). Mr. ChatGPT goes to Washington: OpenAI CEO sam altman testifies before congress on AI risks. Available online at: https://edition.cnn.com/2023/05/16/tech/sam-altman-openai-congress/index.html (accessed June 20, 2023).

[20] Metz C. ‘The Godfather of A.I.' Leaves Google and Warns of Danger Ahead. (2023). Available online at: https://www.nytimes.com/2023/05/01/technology/ai-google-chatbot-engineer-quits-hinton.html (accessed June 20, 2023).

[21] Sadowski J Viljoen S Whittaker M. Everyone should decide how their digital data are used—not just tech companies. Nature. (2021) 595:169–171. 10.1038/d41586-021-01812-3 34211184

[22] Frankfurt HG. On bullshit. Raritan Q Rev. (1986) 6:81–100.

[23] Frankfurt HG. On Bullshit. Princeton, NJ: Princeton University Press (2005).

[24] Hoppenstedt M,. Chefin der Signal-App: Wir werden nicht auf den KI-Zug aufspringen. Interview with Meredith Whittaker. (2023). Available online at: https://www.spiegel.de/netzwelt/netzpolitik/signal-chefin-meredith-whittaker-wir-werden-nicht-auf-den-ki-zug-aufspringen-a-0b223227-560a-41f9-b772-7d7860df3098 (accessed June 20, 2023).

[25] Price WN Gerke S Cohen GI. Potential liability for physicians using artificial intelligence. JAMA. (2019) 322:1765–6. 10.1001/jama.2019.15064 31584609

[26] Haupt CE Marks M. AI-generated medical advice-GPT and beyond. JAMA. (2023) 329:1349–50. 10.1001/jama.2023.5321 36972070

[27] Orwell G. Nineteen *Eighty-Four*. London: Martin Secker and Warburg Ltd (1949).

[28] Lima C,. AI chatbots Won't Enjoy tech's Legal Shield, Section 230 Authors Say. (2023). Available online at: https://www.washingtonpost.com/politics/2023/03/17/ai-chatbots-wont-enjoy-techs-legal-shield-section-230-authors-say/ (accessed June 20, 2023).

